# Temperature-tuned ferromagnetism in hydrogenated multilayer graphene†

**DOI:** 10.1039/c8ra02648c

**Published:** 2018-04-09

**Authors:** Man Zhao, He Xiao, Shuai Chen, Tianjun Hu, Jianfeng Jia, Haishun Wu

**Affiliations:** Key Laboratory of Magnetic Molecules & Magnetic Information Materials Ministry of Education, Shanxi Normal University Linfen China 041004 xiaohe200808@sxnu.edu.cn jiajf@dns.sxnu.edu.cn; The School of Chemical and Material Science, Shanxi Normal University No. 1, Gongyuan Street Linfen China 041004; State Key Laboratory of Coal Conversion, Institute of Coal Chemistry, Chinese Academy of Sciences Taiyuan China 030001

## Abstract

Improving the ferromagnetism properties of pure carbon-based materials is extremely important for their application in spintronics. Hydrogenation of graphene is an effective way to induce magnetic moment into graphene with the advantage of reversibility. However, little experimental work has been done to prove the effect of hydrogen on the magnetic properties of graphene so far, except for systems containing a large amount of oxygen or plasma-induced vacancy which complicated the magnetic origin. Here we report a facile electrochemical cathodic method to generate hydrogenated multilayer graphene or few-layer graphite using graphite powder as the raw material, and observed hydrogen-induced ferromagnetism in samples annealed at different temperatures. The observed results suggest that ferromagnetism of hydrogenated multilayer graphene can be tuned by high temperature treatment, which is attributed to a changeable relative amount of hydrogen atoms chemisorpted on two different sublattices during thermal treatment.

## Abstract 2

Hydrogenated multilayer graphene was prepared by a facile electrochemical cathodic method and its ferromagnetism was tuned through thermal annealing.

## Graphical abstract

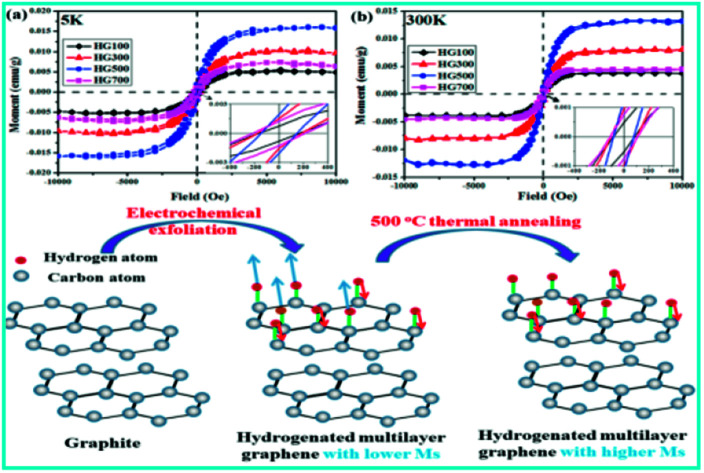

## 1. Introduction

Hydrogenation of graphene has attracted vast attention recently due to the possibility of tuning its mechanical, electronic, and magnetic properties in a reversible way. For instance, graphene and graphane (fully hydrogenated graphene) have been shown to be diamagnetic, while semi-hydrogenated graphene is predicted to be ferromagnetic.^1,2^ Not only theoretically, experimental results also prove that hydrogen atoms on graphene could induce magnetic moments and these moments can couple ferromagnetically over a long distance, making hydrogenated graphene a promising candidate for application in spin electronics.^3^ However, there are two issues to be solved before this dream comes true. One is the requirement of high quality hydrogenated graphene. Magnetic moments induced by methods such as formation of defects and vacancies make the magnetic properties in hydrogenated graphene more complicated.^4–7^ Introducing a magnetic moment into graphene is easy to operate, but it's difficult for these magnetic moments to couple in a ferromagnetic order. Due to the biparticle nature of the graphene lattice, hydrogen located on the sites of the same sublattice should couple ferromagnetically, otherwise on different sublattice should be non-magnetic or antiferromagnetic,^8^ basically following the rules obtained from Lieb's theorem.^9^ However, from the experimental point of view, the hydrogen adsorption process is blind to the graphene lattice, which explained the low magnetism we could obtain regardless of the high defect degree on the graphene surface.^10–12^ While it is worth mentioning that it is now practically feasible to control the adsorption and desorption of single hydrogen atoms on the surface of graphene with new developments in techniques, such as STM imaging and feedback-controlled lithography,^13,14^ but these methods can't be extended to industrial scales. It is also suggested theoretically preparing semi-hydrogenated graphene from graphane using physical method, such as applying an external electric field^15^ or applying pressure on a fluorinated BN sheet supported on a graphane sheet.^16^ Unfortunately, these methods have not been experimentally demonstrated. Thus, controlling the adsorption of hydrogen atoms on the same sublattice of graphene by macroscopic method still remains a big challenge to achieve high saturation magnetization in carbon-based materials.

Unlike the monolayer graphene, for multilayer graphene or few-layer graphite, theoretical studies show that the typical Bernal stacking of graphite planes effectively breaks sublattice symmetry, making two sublattices inequivalent from each other,^17,18^ which reminds us to use hydrogenated multilayer graphene or few-layer graphite instead of few-layer graphene or single layer graphene as sample to tailor the magnetic properties of graphenic materials.

Here we report an electrochemical cathodic method to generate hydrogenated multilayer graphene (few-layer graphite) based on previous work,^19^ but this time we use graphite powder as raw material, which is much cheaper compared with highly oriented pyrolytic graphite. By reducing graphite, the sample we achieved is almost free of structural vacancy and any other defects usually caused by oxidation. If these defects also contribute to the magnetic moments, misinterpretation of the results might occur. To that extent, our sample could be a better candidate to study the magnetic properties of hydrogenated multilayer graphene. Then the superconducting quantum interference device (SQUID) is utilized to measure its magnetic properties. The hysteresis loop measurement of the sample shows an obvious ferromagnetism signal at room temperature, more interestingly, our study reveals that the magnetism doesn't decrease when annealed at elevated temperature. Annealing at a certain temperature is helpful to achieve higher magnetism. FTIR, TG-MS results suggest that different desorption rates of hydrogen atoms bonded on different sublattices through thermal annealing process is the main reason.

## 2. Experiment

### 2.1 Chemicals and materials

Graphite flake powder (80 mesh) was purchased from Qingdao Graphite Company. Tetrabutylammonium hexafluorophosphate (TBAPF_6_, 99%, powder) was obtained from Sigma-Aldrich and used as received. Propylene carbonate (99.9%) was got from Aladdin.

### 2.2 Preparation of hydrogenated multilayer graphene

Graphite powder (40 mg) was put in a porous plastic tube with a platinum plate inserted as negative electrode, another platinum plate served as positive electrode. Both electrodes were immersed in propylene carbonate (PC) with tetra-butylammonium hexafluorophosphate (TBAPF_6_; 0.1 M) and a voltage of 30 V was applied for 12 h. After the electrochemical expansion step, the expanded powder was collected, and afterwards thoroughly washed with a mixture of propylene carbonate and ethanol several times by alternating first dissolving and then separation through centrifugation (10 000 rpm, 5 min). After the washing step, the product was collected by vacuum filtration.

### 2.3 Characterization

X-ray diffraction (XRD) analysis were carried out on an X-ray diffractometer (Ultima IV-185) with Cu Kα radiation. Raman spectra were recorded on a Jobin Yvon LabRAM HR800 micro-Raman spectrometer using a laser excitation of 514 nm at room temperature. X-ray photoelectron spectroscopy (XPS) was performed on ThermoFisher SCIENTIFIC using Al Kα X-ray source. Magnetization measurements were performed using a Quantum Design MPMS XL-5 superconducting quantum interference device (SQUID) magnetometer. The morphologies of the samples were investigated by transmission electron microscopy (TEM, FEI Tecnai F20) and scanning electron microscopy (SEM, JSM-7001F). The magnetic impurity elements (such as Fe, Co and Ni) of all the samples were measured by the inductively coupled plasma spectrometry (ICP, Jarrell-Ash, USA). The thermal gravimetry-mass spectrum (TG-MS) measurements were performed under a flow of argon (30 ml min^−1^) using an Evolution and OMNI star equipment. Fourier transform infrared (FTIR) spectra were recorded using a Bruker spectrometer. Room temperature electron paramagnetic resonance (EPR) spectra were recorded using a Bruker A300 X-band (*m* = 9.4 GHz) spectrometer.

## 3. Results and discussion

As in our previous research, graphite powder was negatively charged by a relatively high voltage. Trace amounts of water even in aprotic solvents were sufficient as a proton donor to observe hydrogenation of aromatic compounds, therefore no dedicated proton donor is typically required.^19^ After twelve hours' electrochemical expansion process in propylene carbonate with tetrabutylammonium hexafluorophosphate (TBAPF_6_) serving as supporting electrolyte, the expanded graphite was subsequently washed and then annealed. HG100, HG300, HG500 and HG700 samples were obtained by annealing the expanded graphite at 100 °C, 300 °C, 500 °C, 700 °C respectively.

TEM images of the samples were first got and illustrated in Fig. S1.† HG300, HG500 and HG700 all exhibit the same typical crumpled structure of electrochemical exfoliated graphene flakes.^20^ This is also supported by the corresponding SEM images which show the wrinkled porous morphologies. One exception is that for HG100, TEM image appears blurred (Fig. S2†). This can be explained by the insertion of solvent into graphite layers, and evidenced by later FTIR results.

**Fig. 1:**
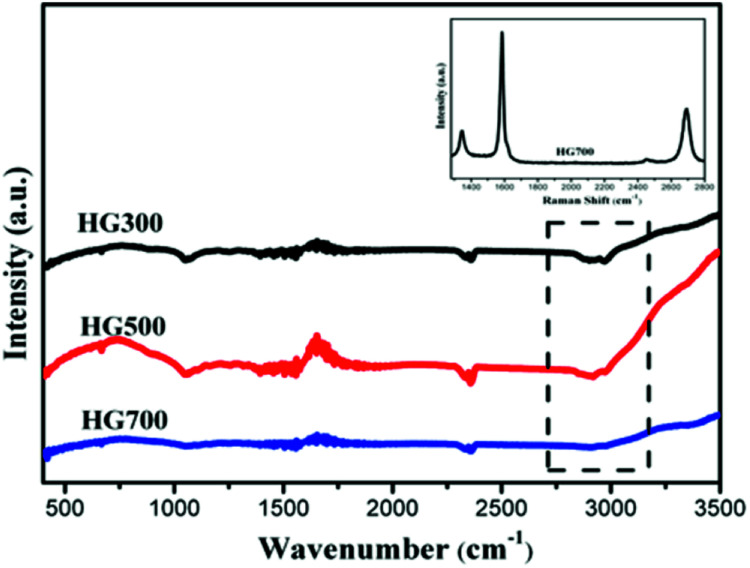
FTIR spectra of HG300, HG500 and HG700. Inset in the Raman spectra of HG700.

**Fig. 2:**
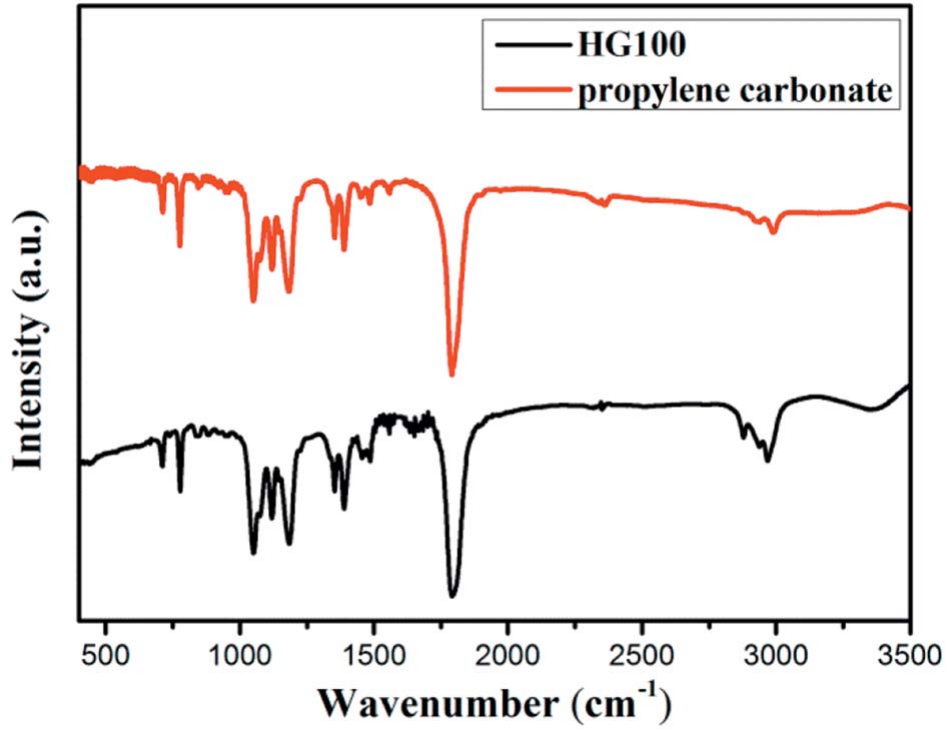
FTIR spectra of HG100 and PC (carbonate propylene).

To determine whether hydrogenation of multilayer graphene (few-layer graphite) is successful, the FT-IR spectra are subsequently employed to identify the presence of chemical bonded hydrogen (Fig. 1). For HG300 and HG500, the evident peaks between 2850 and 2960 cm^−1^ are assigned to the stretching modes of H bonded to an olefinic carbon, which is a clear indication for the formation of hydrogenated graphene.^21^ The fact that the intensity of C–H bond for HG500 is slightly lower than that for HG300 means the lower hydrogenation degree for HG500 in comparison with HG300. With the temperature increasing to 700 °C, the intensity of C–H peaks decreased significantly, indicating that the hydrogen detached from graphene with high temperature, which is in agreement with previous study.^26^ We have also measured the IR spectra of HG100, however, the spectra is almost the same as the spectra of solvent PC (even after extensive washing with benzene, Fig. 2), this is due to the formation of expanded graphite with propylene carbonate inserted into graphite layers in the electrochemical process. During the process of high-temperature thermal annealing, gaseous species generated from decomposition of trapped solvent give another driving force for the graphite exfoliation.^22^

In order to obtain information about the crystal integrity of these flakes, Raman spectra of HG700 is recorded, as shown in the inset of Fig. 1. The intensity ratio of the defect (D) band *versus* the G band is 0.26 in spectra of one typical flake, which is much smaller than the reduced graphene oxide (the *I*_D_/*I*_G_ is much larger than 1).^23^ Considering the large amount of flake edges and residue chemisorbed hydrogen, which can also induce a D peak, it can be concluded that the amount of defect in the sample after high temperature annealing is very low and the crystal structure is almost perfect.

**Fig. 3:**
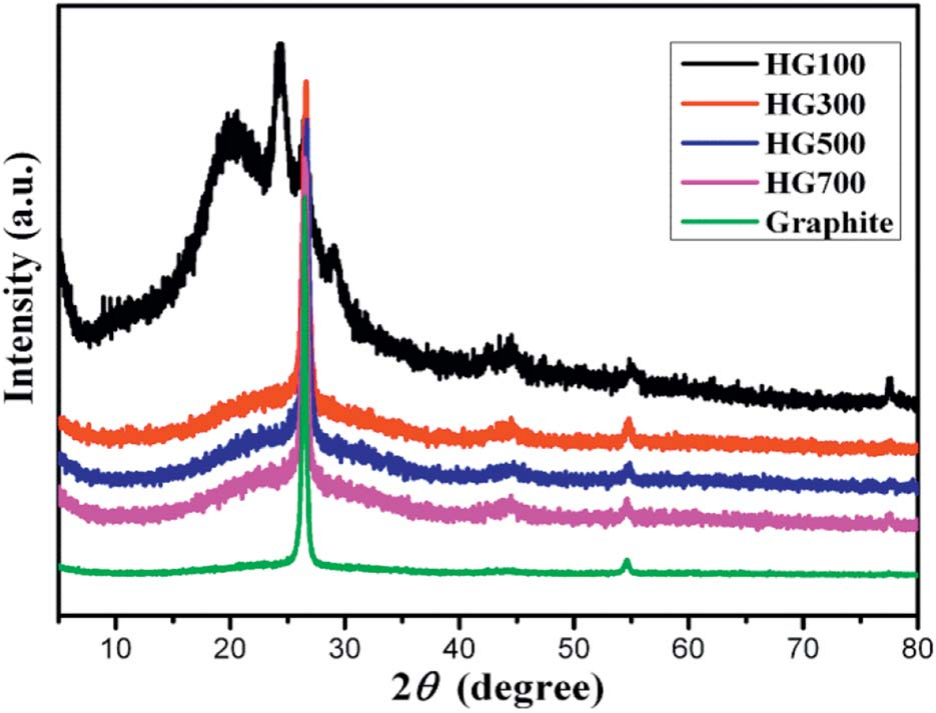
XRD pattern of HG100, HG300, HG500, HG700 and graphite.

X-ray diffraction patterns are displayed in Fig. 3. The XRD pattern of the graphite (black line) exhibits a sharp diffraction peak at 2*θ* = 26.5°, corresponding to the (002) crystal planes of graphite with a layer distance of 3.4 Å.^24^ For HG100, the XRD pattern (green line) exhibits broad new peaks at 2*θ* = 20.3° and 24.2°, indicating an interplanar spacing of 4.4 Å and 3.7 Å. The appearance of these two peaks can be attributed to the inserted solvent propylene carbonate and the covalently bounded hydrogen atoms on the surface of the exfoliated multilayer graphene. With the temperature increased to 300 °C, the intensity of the peak at 2*θ* = 20.3° decreased sharply due to the decomposition and evaporation of solvent. For HG300, HG500, HG700, their XRD patterns exhibit almost the same peaks, a broad peak around 24.2°, and a sharp peak around 26.5°. The former peak originates from the hydrogen atoms covalently bonded on graphene, while the latter peak comes from the crystal structure of graphite, meaning the formation of multilayer graphene with the same stacking order of graphite. Additionally, the amount of hydrogen has no influence on the peak position for HG300, HG500 and HG700 may be due to the curvalete nature of graphene produced by electrochemical method. (Graphene is so curved that hydrogen on the surface doesn't influence the layer distance anymore).

**Fig. 4:**
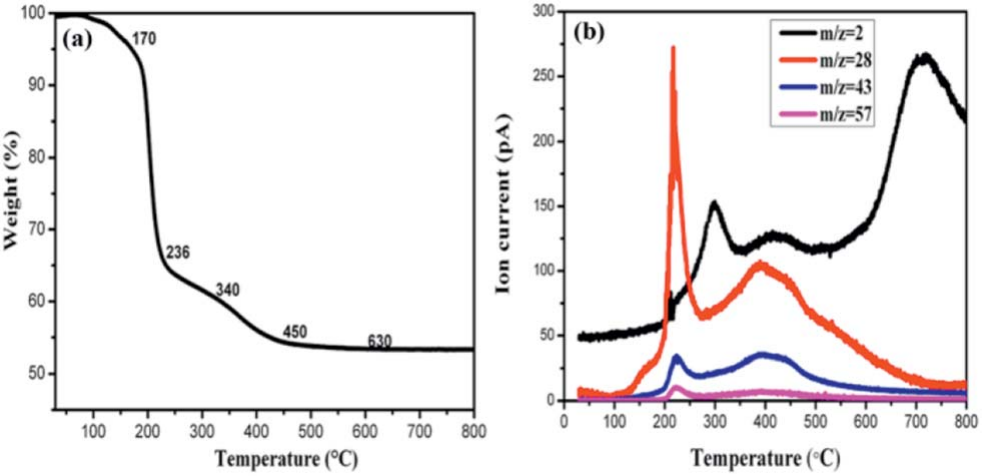
(a) TG profile of HG100 and (b) corresponding MS traces for *m*/*z* = 2 (H_2_), 28, 43, 57 (carbonate propylene).

In order to understand the composition change during the process of thermal annealing, thermogravimetric analysis coupled with mass spectrometry (TG-MS) has been applied. For this purpose, the samples were heated from room temperature to 800 °C under a constant flow of argon and the thermally detached parts were analyzed by an EI mass spectrometer. As depicted in Fig. 4(a), four steps of mass loss, namely region A: 170–230 °C, region B: 230–340 °C, region C: 340–480 °C and region D: 480–790 °C can be identified respectively. The first step can be attributed to molecular fragments exhibiting *m*/*z* = 28/43/57, which are due to residues of intercalated propylene carbonate. Moreover, the amount of propylene carbonate intercalated into graphite is fairly high, accounting for 35% in weight. In the second region B and the fourth region D, the observed mass loss of about 5.8% and 0.5%, can be attributed to the dehydrogenation of the sample as the mass spectrometric analysis exhibits a peak (280 °C, 693 °C) for *m*/*z* = 2 in these two temperature ranges. The hydrogenation degree calculated reaches almost 80%. It should be mentioned that this value is not so accurate as both hydrogen and fragments of propylene carbonate evolved in the temperature region B. In comparison with other hydrogenated graphene whose dehydrogenation occurs mainly at 500 °C,^24,25^ our hydrogenated multilayer graphene or few-layer graphite presents two dehydrogenation peaks, a similar observation was also made in the case of hydrogenated graphene on Ni(111),^26^ they attributed the complex desorption process to different hydrogenation degree. Desorption peak shifts toward lower temperature with higher hydrogenation degree. Besides the high hydrogenation degree, there might be other reasons for this complicated desorption peaks here. Considering the inequality of two sublattices in multilayer graphene (few-layer graphite), the dehydrogenation would occur on two sublattices at different rates.^27^ As a result, the sample will lose hydrogen from one of the sublattice more easily than from the other one, forming two different desorption peaks.

**Fig. 5:**
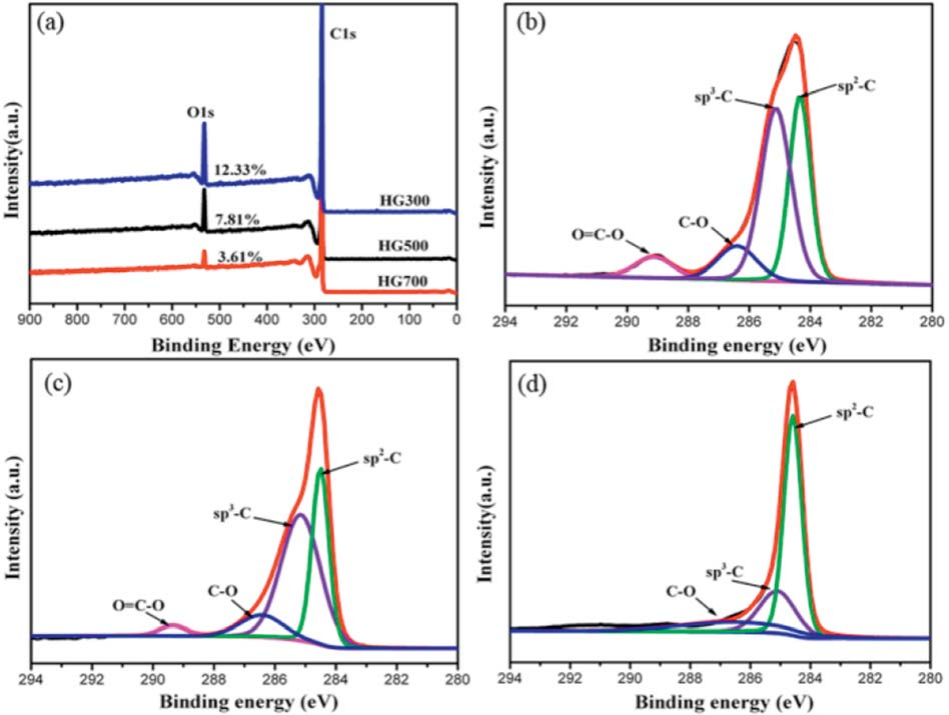
(a) XPS survey spectra for HG300, HG500 and HG700. (b–d) C1s spectra for HG300, HG500 and HG700.

To further elucidate the hydrogen contents and functionalities of the electrochemical reduced hydrogenated multilayer graphene, both wide-scan and high-resolution XPS spectra are performed. As shown in Fig. 5(a), two prominent peaks corresponding to C1s and O1s atomic orbits are observed in the wide-scan spectra for all three samples. With the increase of thermal treatment temperature, the oxygen content decreased from 12.33% to 7.81% for HG300, and to 3.61% for HG700, this is mainly caused by the evolution of propylene carbonate. The C1s spectra of HG300 can be fitted by four peaks (Fig. 5(b)), the main peak located at 284.3 eV corresponds to sp^2^ hybridized carbon atoms, the secondary peak at 285.1 eV is attributed to sp^3^ hybridized C atoms or carbon atoms bond to hydrogen,^28^ the high ratio of sp^3^ C/sp^2^ C means the efficient hydrogenation of the multilayer graphene flakes. The efficient hydrogenation also causes the binding energy shift of the sp^2^ hybridized carbon atoms, from the normal 284.6 eV for HG700 shifts to 284.3 eV for HG300, this new emerged peak corresponding to unhydrogenated carbon atoms next to the C–H bond.^29^ The third and fourth peak located at 286.4 eV and 289.1 eV are assigned to carbon atoms with –C–O and O=C–O bond, respectively. Giving the reducing atmosphere where the sample is generated, these remaining oxygen-containing groups probably stemmed from solvent residues. As evidenced in Fig. 5(c and d), with the increase of thermal treatment temperature, the trapped solvent continue decomposing, as a result the O=C–O peak decreased gradually, and disappeared after 700 °C thermal treatment. Besides the O=C–O peak, the sp^3^ C/sp^2^ C ratio also decreased gradually from HG300 to HG700, this is due to the detachment of hydrogen from graphene surface, as evidenced in previous shown TG-MS and FTIR results.

**Fig. 6:**
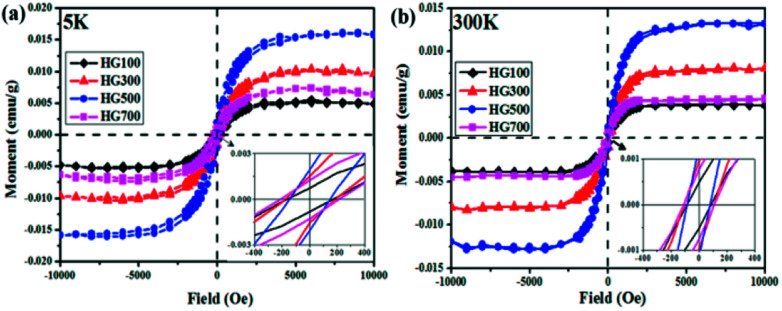
*M*–*H* curves for HG100, HG300, HG500 and HG700 at 5 K (a) and room temperature (b) after subtracting the high field linear background. The insets are the zoom-in part of the hysteresis loops which reveals the soft magnetic character of the hydrogenated graphite.

**Fig. 7:**
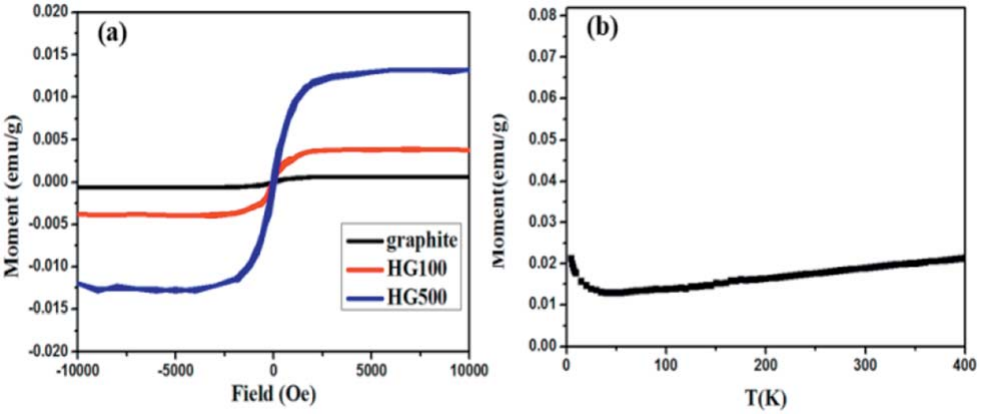
(a) *M*–*H* curves for HG100 and graphite at 300 K; (b) temperature dependent magnetization of the HG500 at a field of 1 T.

Lastly, the superconducting quantum interference device (SQUID) is employed to measure the magnetic properties of the hydrogenated sample treated at different temperatures. Fig. 6(a) shows *M*–*H* curves for all the annealed samples from HG100 to HG700 at 5 K in the field range of −10 kOe < *H* < +10 kOe. The inset in Fig. 6(a) shows a coercivity about 150 Oe, this narrow hysteresis loop suggests a soft magnetic character of GH. In this case, a relatively small magnetic field of 2500 Oe is sufficient for the magnetic saturation of the sample.^10^ After subtracting the high field linear background, the saturation magnetic moment (*M*_s_) of these thermal treated samples are calculated to be 0.005 emu g^−1^ for GH100, 0.01 emu g^−1^ for GH300, 0.016 emu g^−1^ for GH500 and 0.007 emu g^−1^ for GH700, respectively. The magnetization isotherm at 300 K also shows the obvious hysteresis loop for all the samples, meaning the existence of room temperature ferromagnetic state. Compared with 5 K, the *M*_s_ at 300 K is slightly smaller (*M*_s_ = 0.013 emu g^−1^ for HG500) (Fig. 6(b)). While due to the high weight ratio of propylene carbonate in sample HG100, *M*_s_ is significantly smaller than it should be, thus it is not to be compared with saturation magnetization of other annealed samples. With the annealing temperature increased from 300 °C to 500 °C, the *M*_s_ went up sharply. But as we go for HG-700, we observed a sudden fall in the magnetic moment. This is due to the less magnetic moment induced by the hydrogen as more hydrogen atoms desorbed from the surface of sample with higher temperature. Considering the ferromagnetic influence of the staring material, the *M*–*H* curve of graphite is also investigated. As shown in Fig. 7(a), although graphite shows ferromagnetic hysteresis loop, but the *M*_s_ is very low, much lower than the lowest *M*_s_ of HG100. So the impurity in graphite is not the reason for magnetic change in HG100, HG300, HG500 and HG700. Moreover, to further confirm the magnetic state, we carried out the *M*–*T* measurement of the sample HG500 (Fig. 7(b)), the measurement were recorded in a sweep mode over a temperature range from 5 to 300 K in a field of 1 T after cooling in a field of 1 T. This trend is similar to paramagnetic behavior at the beginning, the magnetization decreases until 58 K, then increases slowly with increasing temperature above 58 K. This increment of the magnetization with increasing temperatures for ferromagnetic *M*–*T* curve is also observed in some diluted magnetic semiconductor,^30,31^ and the author attributed this phenomenon to the increment of the electron carrier concentrations. However, considering the ferromagnetic hysteresis loop is observed in 300 K, which shows a coercivity about 200 Oe, we can conclude that the sample shows ferromagnetism with a Curie temperature higher than 300 K.

As for the reason of the highest *M*_s_ for HG500, in correlation with Lieb's theorem, it can be clearly stated that the hydrogen atoms are more populated on one sublattice than on the other for HG500, although the whole hydrogenation degree is lower than HG300 as suggested by TG-MS and XPS results. This can be explained by the different desorption rates of hydrogen adsorbed on different sublattice.

**Scheme 1:**
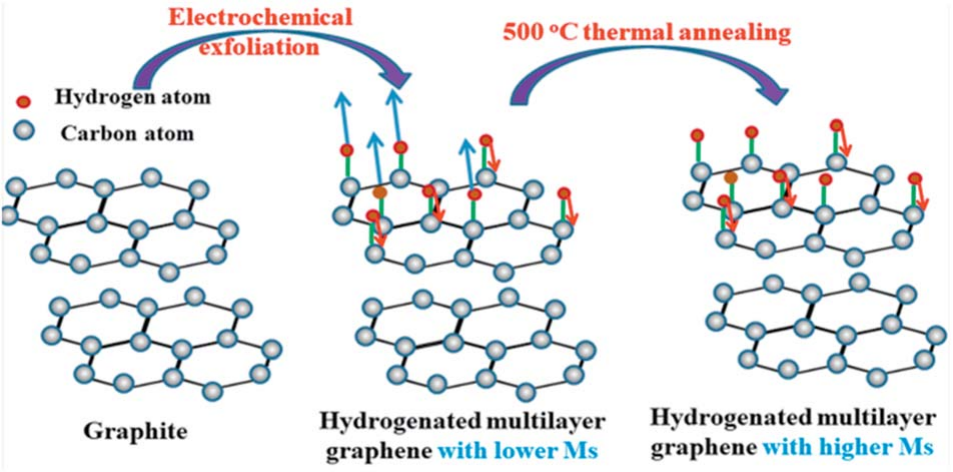
Illustration of temperature-tuned ferromagnetism in hydrogenated multilayer graphene.

When the hydrogenated multilayer graphene (few-layer graphite) is undergoing thermal treatment, the sample will lose H from both sublattices, but at different rates.^27^ In this case hydrogen atoms on one sublattice become more populated than the other. From TG-MS results, it can be concluded that the first dehydrogenation peak in the range of 250–340 °C mainly comes from hydrogen positioned on the same sublattice, and the larger number difference of hydrogen adsorbed on two sublattices contribute to the higher magnetism in HG-500. The effect of temperature on the magnetic property is briefly shown in Scheme 1.

**Fig. 8:**
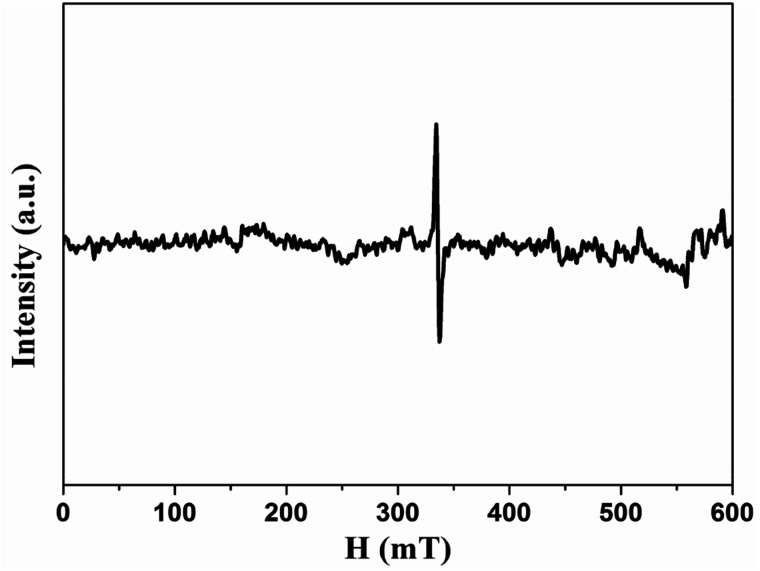
EPR spectra of HG500 at room temperature.

Since the origin of magnetism in pure organic materials has been very controversial, we applied ICP-MS to check the content of possible ferromagnetic impurities in our samples. The total amount of magnetic impurities (Fe, Co, Ni) is 25.2 ppm for HG-700. Given that Fe impurity content of 1 μg g^−1^ would give the magnetization of 2.2 × 10^−4^ emu g^−1^,^32^ the magnetic impurities in HG700 are insufficient to give the observed magnetic moment 0.007 emu g^−1^. Although ICP is rather common, the analyzed amount of magnetic impurities is typically underestimated as not the sample but the components soluble in the standardized nitric acid are detected.^33^ To further explore the origin of magnetism, we conducted EPR measurement. From the EPR spectra (Fig. 8), we observe a signal with a line-width of Δ*H* ≈ 0.7–2.9 mT and a *g* value that is in the 2.006–2.013 range. The small value of the line-width and the small deviation in the *g* value suggest that the unpaired spins do not originate from transition-metal impurities, but probably from carbon-inherited spin species such as defects and edges modified by the hydrogenation.^34^ Furthermore, since all the samples (HG100, HG300, HG500, HG700) come from the same source, the magnetic contribution from the possible metallic impurity should be similar for all these samples. So the smaller magnetic moment of HG-700 suggests that metallic impurity should not be the reason of the observed higher ferromagnetism of HG-500. All the above results suggest that magnetism is the intrinsic nature of hydrogenated multilayer graphene.

## 4. Conclusions

In summary, we proposed a facile method to generate hydrogenated multilayer graphene or few-layer graphite with rare oxygen-containing group bonded on the lattice due to the reducing atmosphere in the electrochemical process. With few magnetic moments induced by other defects, hydrogen is the main cause of magnetism, thus this sample could be a better candidate to study the magnetism of hydrogenated graphene. Then an easy way was applied to tune the magnetic properties of hydrogenated multilayer graphene by annealing at different temperatures. The influence of temperature on magnetism can be attributed to different desorption energies of hydrogen on two inequivalent sublattices. Moreover, this study may also provide clues for the design of functionalized graphene-based samples with sublattice selectivity by macroscopic method.

## Conflicts of interest

There are no conflicts to declare.

## Supplementary Material

RA-008-C8RA02648C-s001
