# Supplementary material for: Temperature-tuned ferromagnetism in hydrogenated multilayer graphene
Source: RSC Adv. 2018 Apr 9;8(24):13148–53. doi: 10.1039/c8ra02648c (PMC9079760; doi:10.1039/c8ra02648c)
Supplement: RA-008-C8RA02648C-s001 [file RA-008-C8RA02648C-s001.pdf]

Electronic Supplementary Material (ESI) for RSC Advances.
This journal is © The Royal Society of Chemistry 2018

Supporting information

Temperature-tuned ferromagnetism in hydrogenated multilayer graphene

Man Zhao,[a,b] He Xiao,*[a,b] Shuai Chen,[c] Tianjun Hu,[a,b] Jianfeng Jia*[a,b] and Haishun Wu[a,b]

[a] *Key Laboratory of Magnetic Molecules & Magnetic Information Materials Ministry of Education, ShanxiNormal University. Linfen, China, 041004.*

[b] *The School of Chemical and Material Science, Shanxi Normal University, No. 1, Gongyuan Street, Linfen, China, 041004*

[c] *State Key Laboratory of Coal Conversion, Institute of Coal Chemistry, Chinese Academy of Sciences, Taiyuan,China,030001.*

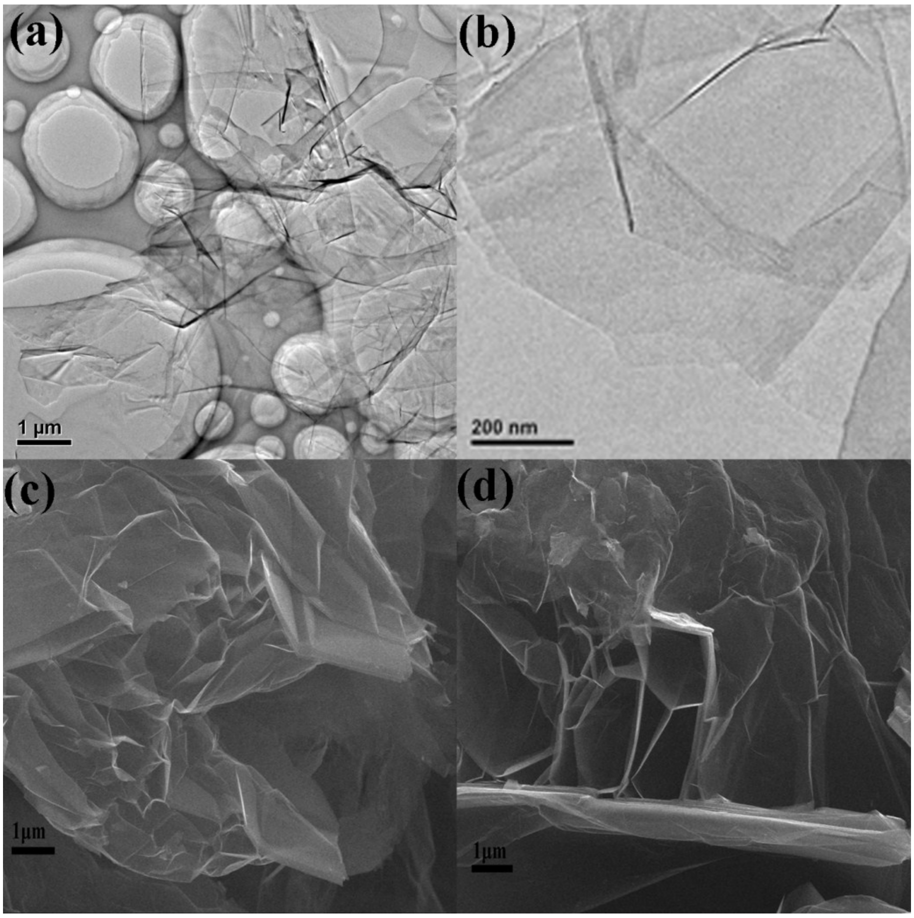

Fig. S1. (a, b) TEM images of HG300. (c, d) SEM images of HG300.

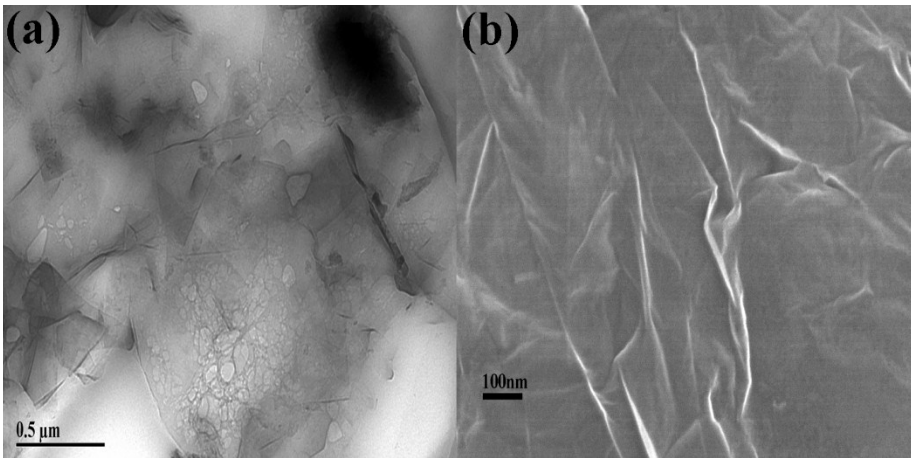

Fig. S2. (a) TEM image of HG100. (b) SEM image of HG100.
